# Supplementary material for: Venomix: a simple bioinformatic pipeline for identifying and characterizing toxin gene candidates from transcriptomic data
Source: PeerJ. 2018 Jul 31;6:e5361. doi: 10.7717/peerj.5361 (PMC6074769; doi:10.7717/peerj.5361)
Supplement: Supplemental Information 6 [file peerj-06-5361-s006.gz › FinalOutput_1E-6/Venom_peptide_SjAPI-2_1/finaltree.pdf]

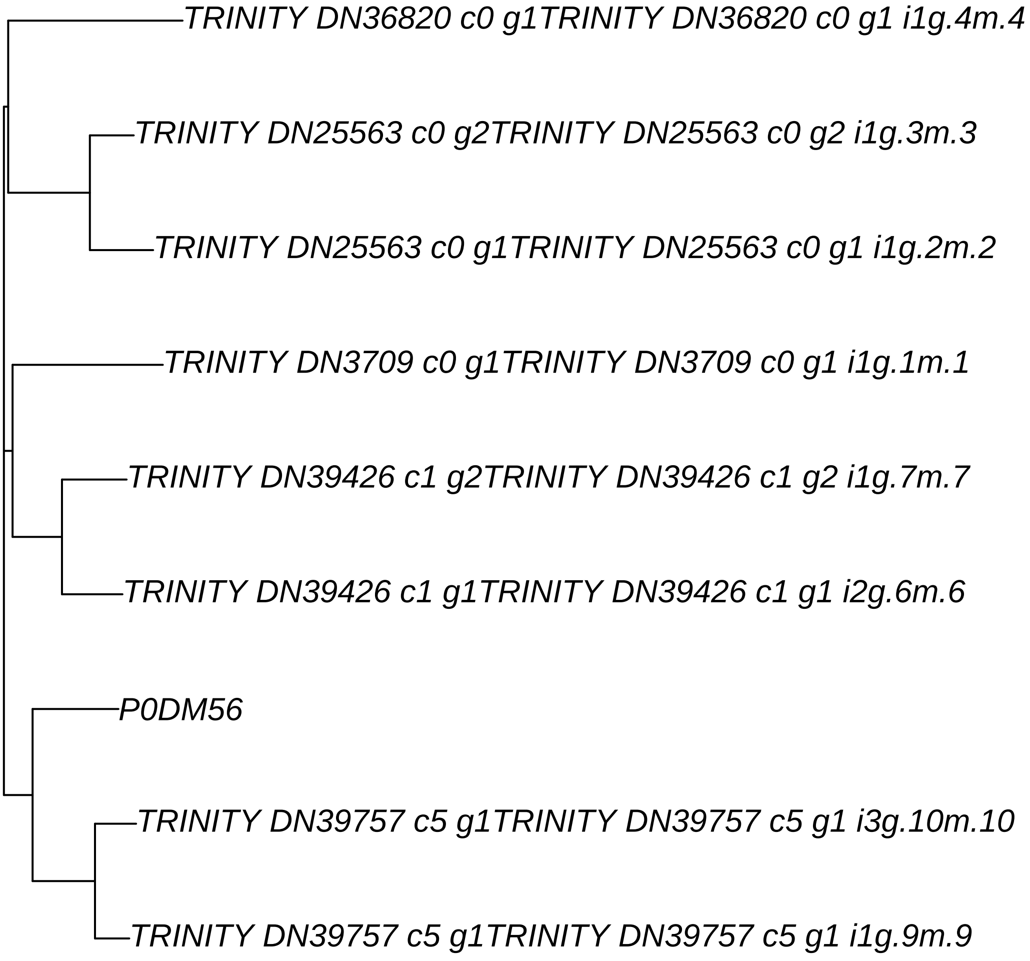
TRINITY DN36820 c0 g1TRINITY DN36820 c0 g1 i1g.4m.4
TRINITY DN25563 c0 g2TRINITY DN25563 c0 g2 i1g.3m.3
TRINITY DN25563 c0 g1TRINITY DN25563 c0 g1 i1g.2m.2
TRINITY DN3709 c0 g1TRINITY DN3709 c0 g1 i1g.1m.1
TRINITY DN39426 c1 g2TRINITY DN39426 c1 g2 i1g.7m.7
TRINITY DN39426 c1 g1TRINITY DN39426 c1 g1 i2g.6m.6
P0DM56
TRINITY DN39757 c5 g1TRINITY DN39757 c5 g1 i3g.10m.10
TRINITY DN39757 c5 g1TRINITY DN39757 c5 g1 i1g.9m.9
